# Gut Microbiome Signatures Distinguish Susceptibility from Disease Development in Type 2 Diabetes

**DOI:** 10.3390/ijms27073160

**Published:** 2026-03-31

**Authors:** Chen Ifrach, Ruth Levy-Turgeman, Amir Szitenberg, Inbar Kesten, Milena Pitashny, Nomy Levin-Iaina, Yael Segev, Yoram Yagil

**Affiliations:** 1 Laboratory for Molecular Medicine, Barzilai University Medical Center, Ashkelon 78278, Israel; cheni@bmc.gov.il (C.I.); nomyl@bmc.gov.il (N.L.-I.); 2 Faculty of Health Sciences, Ben-Gurion University of the Negev, Beer Sheba 8410501, Israel; 3 The Mantoux Bioinformatics Institute of the Nancy and Stephen Grand Israel National Center for Personalized Medicine, Weizmann Institute of Science, Rehovot 7632706, Israel; amir.szitenberg@weizmann.ac.il; 4 Clinical and Research Microbiome Center, Rambam Health Care Campus, Haifa 3109601, Israel; 5 Hillel Yaffe Medical Center, Hadera 3820302, Israel; 6 Faculty of Medicine, Technion Institute of Technology, Haifa 3109601, Israel

**Keywords:** rat models, Cohen diabetic rat, diabetes, microbiome, genus, susceptibility, disease development

## Abstract

Individuals may be prone or resistant to the development of type 2 diabetes. The basis for susceptibility is in part genetic, but environmental factors are likely to come into play. The gut microbiome stands at the interface of genetics and the host microenvironment. Its role in mediating susceptibility to diabetes, however, has not been resolved. Here we investigated whether the gut microbial composition contributes to susceptibility to diabetes, as distinct from disease development. We hypothesized that distinct microbial signatures modulate sensitivity or resistance to a diabetogenic diet (DD) and that separate signatures are linked to disease development. To test this hypothesis, we studied the Cohen diabetic rat model, comprising a diabetes-sensitive strain (CDs/y) and a diabetes-resistant strain (CDr/y). When exposed to DD, diabetes develops in CDs/y but not in CDr/y rats; on a regular diet (RD), both strains remain metabolically normal. To establish the contribution of the gut microbiome to susceptibility, we studied the fecal microbial composition in young, metabolically healthy CDs/y and CDr/y rats, using 16S rRNA gene sequencing, measures of α- and β-diversity, and differential taxonomic abundance. We found distinct, strain-specific gut microbiota profiles that differentiated diabetes-sensitive from -resistant animals, indicating an association between microbial composition and susceptibility. To test causality, we co-housed sensitive and resistant animals to allow passive microbial cross-transfer and fed the animals with DD. Co-housing led to partial convergence of microbial communities and significantly attenuated the diabetic phenotype in CDs/y rats, supporting a contributory and causal role for the gut microbiome in modulating sensitivity to diabetes. The resistance phenotype, on the other hand, remained unchanged. To distinguish between the contribution of the gut microbiome to susceptibility to diabetes as opposed to the development of the disease, we studied the gut microbial profiles across strains after feeding with DD or RD and the development of diabetes in CDs/y but not in CDr/y. We found distinct taxonomic signatures that differentiated diabetic from non-diabetic animals. These findings demonstrate that the gut microbiome contributes to susceptibility to diabetes with separate pathways from those linked to the development of diabetes and may represent an important modifiable determinant of diabetes risk and a target for early intervention.

## 1. Introduction

Type 2 diabetes mellitus is among the most prevalent complex diseases worldwide. Although its pathogenesis has been extensively studied, the determinants of why some individuals develop diabetes whereas others remain disease-free are far less understood. This knowledge gap reflects the limited investigation of individual disease susceptibility, distinct from mechanisms operating during the development of the disease.

Susceptibility denotes differential sensitivity or resistance to diabetogenic triggers operating through specific pathogenetic pathways. Genetic factors contribute substantially, with heritability estimates ranging from 25% to 72% [1], while environmental factors are also critical but remain poorly defined in the context of susceptibility [2]. The gut microbiome, positioned at the interface between host genetics and environmental exposures, has emerged as an important contributor to diabetes pathogenesis [3]. However, its role in determining inherent susceptibility to diabetes prior to its onset remains largely unexplored.

Studies of susceptibility in humans are constrained by genetic, environmental, and phenotypic heterogeneity. Consequently, relatively few human studies have addressed the contribution of the microbiome to disease susceptibility rather than to pathogenesis. Inbred animal models offer important advantages by enabling controlled genetic backgrounds and environmental conditions, thereby facilitating mechanistic investigation of host–microbiome interactions. Nevertheless, few animal models of diabetes permit direct interrogation of susceptibility per se, and such microbiome-based studies remain scarce.

The Cohen model used in the present study uniquely addresses this gap. This fully inbred, non-obese, and validated model of type 2 diabetes recapitulates key features of the human disease [4]. Importantly, the model comprises two closely related strains with divergent phenotypes, a diabetes-sensitive strain (CDs/y) and a diabetes-resistant strain (CDr/y), enabling direct investigation of the mechanisms underlying differential susceptibility.

In this study, we investigated the association between gut microbiome composition and both diabetes susceptibility and development in the Cohen rat model. Our primary objective was to test the hypothesis that gut microbial composition causally influences susceptibility to diabetes in response to a defined dietary challenge. We sought to identify bacterial signatures present during health that predict sensitivity or resistance to disease. Our secondary objective was to examine the relationship between the gut microbial composition and the development of diabetes, which has not been previously addressed in this model, and to determine whether bacterial signatures distinct from susceptibility are involved.

## 2. Results

### 2.1. Experiment 1

#### 2.1.1. Body Weight

**Figure 1:**
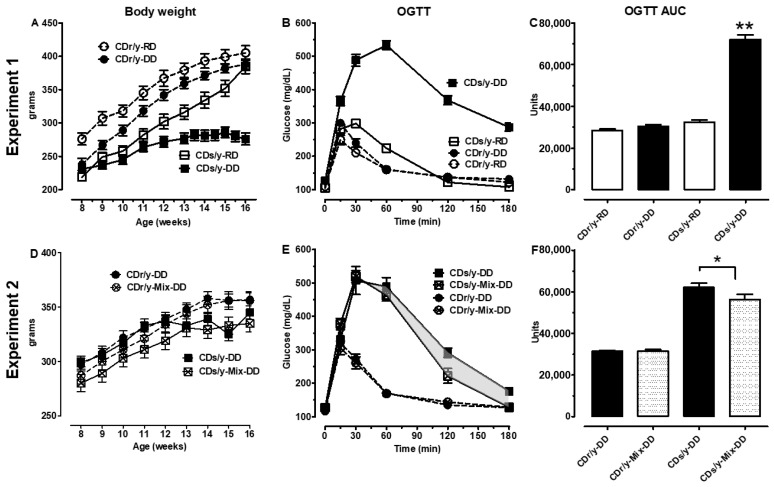
Body weight and metabolic profile. Experiment 1: Body weight (**A**) and oral glucose tolerance test (OGTT) (**B**) with the corresponding area under the curve (AUC) (**C**) in CDr/y and CDs/y fed RD or DD (n = 9 in each group). Experiment 2: Body weight (**D**) and OGTT (**E**) with the AUC (**F**) in CDr/y and CDs/y fed DD (control groups; n = 6 in each group) and after co-housing in CDr/y-Mix-DD and CDs/y-Mix-DD (n = 8 in each group). The shaded area in (**E**) indicates the difference in the OGTT response between CDs/y-DD and CDs/y-Mix-DD, reflecting the effect of co-housing. Data are presented as mean ± SEM. * *p* < 0.01, ** *p* < 0.001 by one-way ANOVA and Fisher’s LSD post hoc test.

Body weight increased similarly in CDr/y-RD, CDr/y-DD, and CDs/y-RD (Figure 1A). In contrast, CDs/y-DD gained less weight during the initial weeks of the experiment compared to the other groups, and reached a plateau by week 4, coinciding with diabetes onset. This growth trajectory is consistent with previous reports on this model [4].

#### 2.1.2. Glucose Tolerance

At baseline, whole blood glucose concentrations were similar across all four groups (Figure 1B). Following glucose loading, CDs/y-RD, CDr/y-RD and CDr/y-DD exhibited a rapid increase in glucose, peaking within 15–30 min and returning to baseline within 2 h, consistent with preserved glucose tolerance. In contrast, CDs/y-DD showed a delayed glucose rise, peaking at 60 min, with an incomplete return to baseline thereafter. Accordingly, the AUC did not differ among the three control groups but was significantly increased in CDs/y-DD compared with all other groups (*p* < 0.001, one-way ANOVA, F = 242), indicating markedly impaired glucose tolerance.

#### 2.1.3. Characterization of the Gut Microbiome

We profiled the fecal microbiome across experimental groups to assess how host strain, age, and diet influence the gut bacterial community. Analyses focused on: (a) strain-specific differences in microbial composition between CDs/y and CDr/y animals maintained on an RD at two ages; (b) age/time-related changes in microbial profiles within each strain; and (c) the impact of dietary intervention on microbial composition. After quality filtering, samples had a median sequencing depth of ~24,000 reads. All samples were retained following rarefaction to 12,000 sequences per sample, yielding 3040 ASVs spanning 13 bacterial phyla.

#### 2.1.4. Effect of Strain

To evaluate the influence of host genetic background on gut microbiome diversity, we compared CDs/y and CDr/y strains maintained on RD at 8 weeks (n = 18 per strain) and 16 weeks of age (n = 9 per strain).

**Figure 2:**
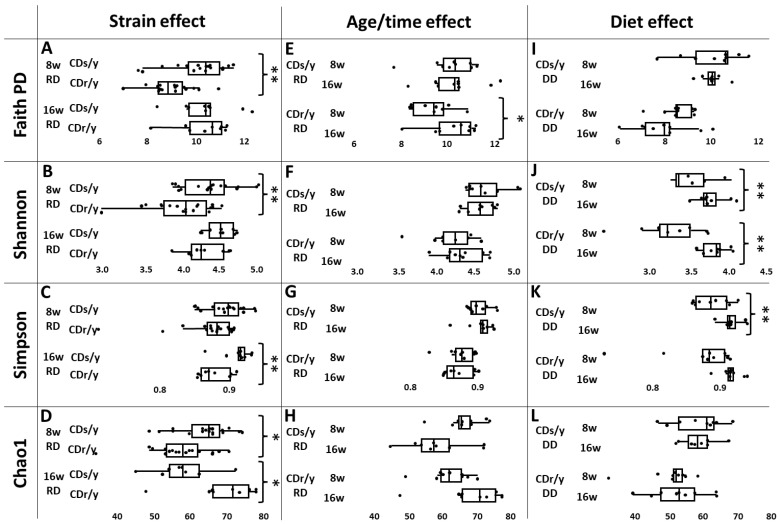
Effects of strain, age/time and diet on α-diversity. We analyzed α-diversity using 4 indices: Faith PD (**A**,**E**,**I**), Shannon (**B**,**F**,**J**), Simpson (**C**,**G**,**K**) and Chao1 (**D**,**H**,**L**). In “Strain effect” analysis, n = 18 in each group; in age/time and diet effect analyses, n = 9 in each group. Data are presented as box-and-whisker plots with the median indicated by the center line, boxes representing the interquartile range (Q1–Q3) and whiskers extending to 1.5 IQR from the quartiles. Between-group comparisons were performed by one-way ANOVA, * *p* < 0.05 ** *p* < 0.001. w, weeks.

α-diversity (Figure 2A–D and Table 1): At 8 weeks, CDs/y exhibited higher gut microbiome richness and evenness compared to CDr/y across multiple indices. By 16 weeks, these differences were reduced, except for Chao1 richness, which remained higher in CDr/y, indicating age-dependent and strain-specific changes in α-diversity patterns.

**Figure 3:**
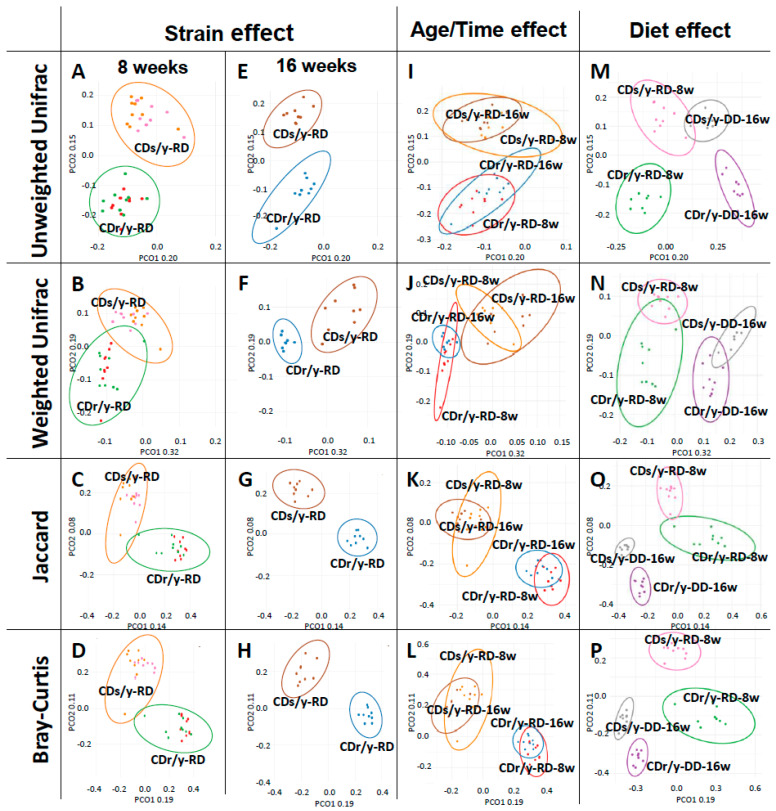
Effects of strain, age/time and diet on β-diversity. We analyzed β-diversity using 4 indices: unweighted Unifrac (**A**,**E**,**I**,**M**), weighted Unifrac (**B**,**F**,**J**,**N**), Jaccard (**C**,**G**,**K**,**O**) and Bray–Curtis (**D**,**H**,**L**,**P**). Each point represents an individual sample; group clustering is indicated by enclosing circles. In “Strain effect” analysis, n = 18 in each group; in age/time and diet effect analyses, n = 9 in each group. Statistical comparisons between groups were performed by PERMANOVA and are reported in Table 1. w, weeks.

β-diversity (Figure 3A–H and Table 1): We observed distinct strain-specific clustering of microbial communities at both 8 and 16 weeks, indicating a consistent age/time-independent effect of host strain on community composition.

#### 2.1.5. Effect of Age/Time

We next assessed age/time-related changes in the gut microbiome by comparing microbial communities at 8 and 16 weeks of age within each strain (CDs/y and CDr/y; n = 9 per group).

α-diversity (Figure 2E–H and Table 1): In CDs/y, α-diversity did not change significantly with age/time. CDr/y showed an increase from 8 to 16 weeks in only Faith’s phylogenetic diversity.

β-diversity (Figure 3I–L and Table 1): We observed significant age/time-related shifts in β-diversity in both strains, with clear separation between 8- and 16-week-old animals, indicating that age/time influences microbial community composition irrespective of host strain.

#### 2.1.6. Effect of Diet

To assess the impact of dietary intervention, we examined changes in gut microbiome composition following the switch from RD to DD in CDs/y and CDr/y (n = 9 per group). As feeding CDs/y with DD led to the development of overt diabetes, our ability to distinguish diet-specific effects from diabetes-associated changes was limited in this strain.

α-diversity (Figure 2I–L and Table 1): Dietary effects were strain- and index-dependent. While no significant changes were observed in Faith’s phylogenetic diversity or Chao1 richness, Shannon and Simpson indices increased in CDs/y after the switch to DD, suggesting inconsistent effects of diet on microbial evenness across indices.

β-diversity (Figure 3M–P and Table 1): In contrast, β-diversity analyses revealed pronounced diet-associated shifts in microbial community composition. All β-diversity metrics consistently separated RD- and DD-fed animals, indicating substantial restructuring of the gut microbiome following dietary intervention.

#### 2.1.7. Effect of Interaction of Age/Time and Diet on Diversity

**Figure 4:**
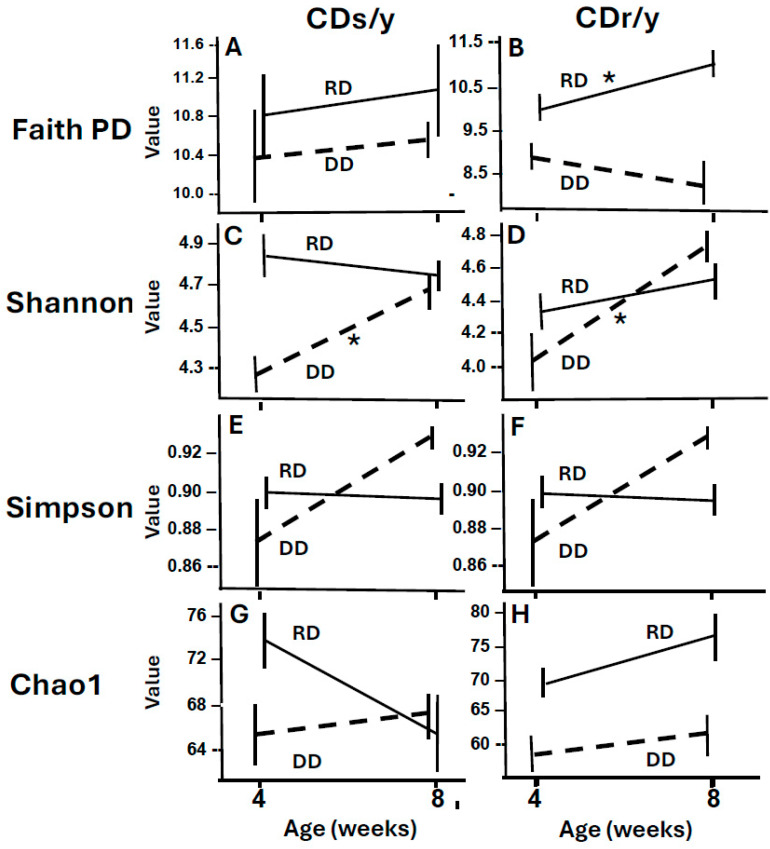
Effect of interaction of age/time and diet on α-diversity. We analyzed α-diversity using mixed-effects models with repeated measures and four indices: Faith PD (**A**,**B**), the Shannon index (**C**,**D**), Simpson (**E**,**F**) and Chao1 (**G**,**H**). For each strain, RD (solid line) was compared with DD (dashed line). Each point represents the mean ± SEM. n = 9 in each group. * *p* < 0.01 for age/time x diet interaction effects.

We identified through mixed-effects analyses strain- and index-dependent interactions between age/time and diet (Figure 4). In CDs/y, Faith’s PD remained stable across age/time and dietary conditions, whereas Shannon and Simpson indices exhibited strong age/time–diet interactions under DD, indicating pronounced changes in community evenness under this dietary condition. Chao1 richness showed only borderline age-related effects under RD. In contrast, in CDr/y animals, Faith’s PD displayed a significant age/time–diet interaction under RD but not under DD, while Shannon and Simpson indices showed weaker yet detectable interactions under DD, consistent with more modest age/time- and diet-associated shifts in diversity. Overall, these results demonstrate that the effects of interaction between age/time and diet on α-diversity depend on host strain, dietary condition, and the diversity metric examined.

#### 2.1.8. Taxonomic Signature Associated with Susceptibility to Diabetes

**Figure 5:**
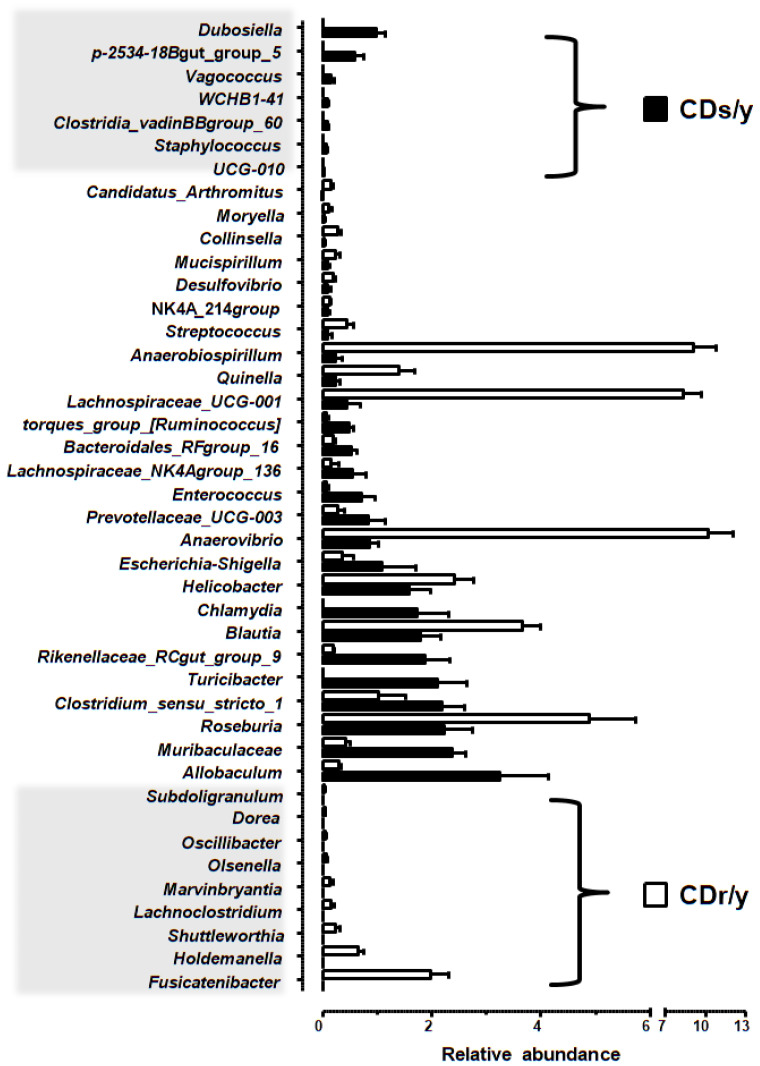
Genus-level microbial signatures associated with susceptibility. Genus-level gut microbial signatures at 8 weeks of age in metabolically healthy Cohen diabetic rats provided with RD (n = 18 in each group). Genera associated with diabetes sensitivity are defined as those significantly enriched in the diabetes-sensitive strain (CDs/y) and not detected in the diabetes-resistant strain (CDr/y) (shaded upper left region). Genera associated with diabetes resistance are defined as those significantly enriched in CDr/y and not detected in CDs/y (shaded lower left region). Genera detected in both strains but differing significantly in relative abundance between CDs/y and CDr/y are also shown. Data are presented as mean ± SEM of relative abundance. Differential abundance between groups was assessed using ANCOM-BC2 (q < 0.05).

At 8 weeks of age, CDs/y and CDr/y exhibited distinct genus-level microbial signatures (Figure 5, Table S1). Seven genera were uniquely enriched in CDs/y, whereas nine genera were uniquely enriched in CDr/y. In addition, twenty-six genera shared between strains differed significantly in relative abundance. Together, these differences indicate the presence of strain-specific genus-level microbial signatures associated with differential susceptibility to diabetes.

#### 2.1.9. Taxonomic Signature Associated with the Development of Diabetes

**Figure 6:**
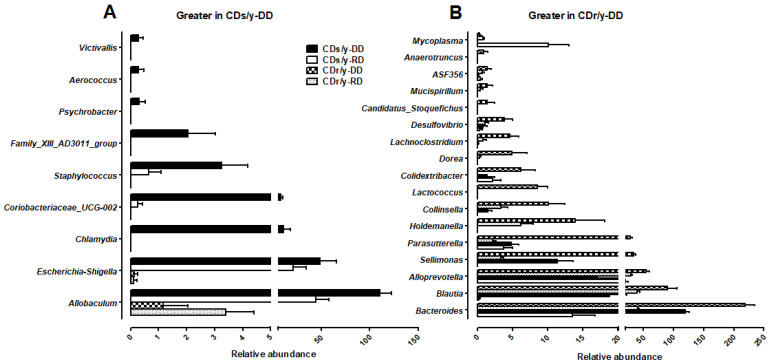
Genus-level microbial signatures associated with development of diabetes. Genus-level gut microbial profiles of diabetes-sensitive (CDs/y) and diabetes-resistant (CDr/y) rats after 8 weeks of feeding with DD or RD at 16 weeks of age (n = 9 in each group). Diabetes developed exclusively in the CDs/y-DD group. (**A**) Genera significantly enriched in CDs/y-DD compared with the three non-diabetic groups (CDs/y-RD, CDr/y-DD and CDr/y-RD). (**B**) Genera significantly enriched in CDr/y-DD compared with the same three groups. Data are presented as mean ± SEM of relative abundance. Differential abundance between groups was assessed using ANCOM-BC2 (q < 0.05).

At 16 weeks, CDs/y (Figure 6A) and CDr/y (Figure 6B) fed DD displayed distinct genus-level microbial signatures compared with the other strain- and diet-matched groups at the same time point. Nine genera were uniquely enriched in CDs/y-DD, whereas seventeen genera were uniquely enriched in CDr/y-DD (Table S1). Together, these differences indicate pronounced strain-specific divergence in genus-level microbial signatures associated with the development of diabetes in CDs/y but not in CDr/y following DD.

### 2.2. Experiment 2

#### 2.2.1. Body Weight

Body weight increased over time in all experimental groups and did not differ significantly between single-housed and co-housed animals (Figure 1D).

#### 2.2.2. Glucose Tolerance

We assessed the effect of co-housing on glucose tolerance by comparing co-housed CDs/y-Mix-DD (n = 8) with single-housed CDs/y-Ctrl-DD (n = 8), and co-housed CDr/y-Mix-DD (n = 8) with single-housed CDr/y-Ctrl-DD (n = 8) (Figure 1E,F). Baseline glucose levels were comparable across all groups. In CDr/y animals, co-housing did not significantly alter the OGTT compared with single-housed controls. In contrast, in CDs/y, co-housing was associated with an attenuated OGTT curve, reflected by an approximately 10% reduction in the AUC compared with single-housed controls (*p* < 0.01).

#### 2.2.3. Microbiome Analysis

The pre-processed ASV table used for downstream analyses had a median sequencing depth of approximately 33,000 reads per sample. All samples were retained after rarefaction to 12,000 sequences per sample, yielding 3016 unique ASVs spanning 12 bacterial phyla.

Following co-housing, we did not find differences in α-diversity between CDs/y-DD and CDs/y-Mix-DD, nor between CDr/y-DD and CDr/y-Mix-DD (Table 1). In contrast, we detected significant differences in β-diversity between these groups (Table 1), indicating alterations in microbial community composition associated with co-housing in both strains. Consistent with this finding, the pronounced differences in microbial composition observed between CDs/y-DD and CDr/y-DD at 16 weeks were attenuated following co-housing, as reflected by reduced β-diversity separation between CDs/y-Mix-DD and CDr/y-Mix-DD (Table 1).

**Figure 7:**
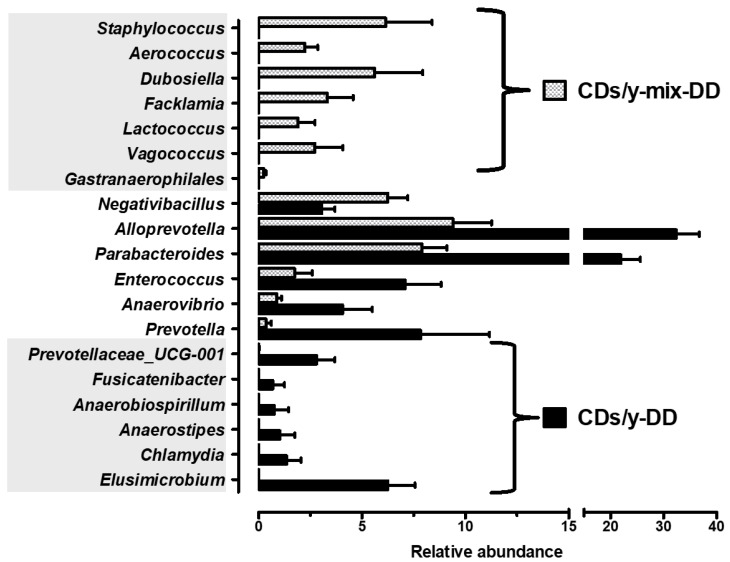
Genus-level microbial signatures in the co-housing experiment. Genus-level microbial signatures of CDs-DD (n = 6; shaded area, lower left) and of co-housed CDs-Mix-DD animals (n = 8; shaded area, upper left). Data are presented as mean ± SEM of relative abundance. Differential abundance between groups was assessed using ANCOM-BC2 (q < 0.05).

Genus-level taxonomic comparisons identified six genera uniquely enriched in CDs/y-DD and seven uniquely enriched in CDs/y-Mix-DD (Figure 7, Table S1), indicating co-housing-associated shifts in genus-level microbial community composition.

## 3. Discussion

In the present study, we investigated the contribution of the gut microbiome to susceptibility to and development of diet-induced diabetes in the Cohen rat model. Comparative analyses revealed distinct population structures in the diabetes-sensitive CDs/y and -resistant CDr/y strains, highlighting a strong association between gut microbiota composition and diabetes susceptibility. Co-housing–mediated passive microbial exchange between resistant and sensitive animals attenuated the diabetic phenotype in the susceptible strain, supporting a contributory role for the gut microbiome in modulating susceptibility to diet-induced diabetes. In addition, we identified specific bacterial signatures associated with the development of the diabetic phenotype in this model.

The primary objective of this study was to determine whether the gut microbiome is involved in susceptibility to diabetes in our rat model, beyond the well-established genetic predisposition [4,5]. While the influence of the gut microbiome on disease pathogenesis has been extensively investigated [3,6], its role in shaping susceptibility remains less well defined. In healthy individuals, the gut microbiome is characterized by high α-diversity and a specific bacterial composition [3,7], whereas dysbiosis represents an early event in diabetes [8]. As disease progresses from health to prediabetes and to overt diabetes, each stage is associated with a distinct microbiome profile [9]. It remains unclear, however, whether specific dysbiotic shifts in microbial composition can serve as predictive markers of disease sensitivity or resistance, as distinct from microbiome changes associated with disease development.

We conducted this study using the Cohen diabetic rat model [4,5], which is uniquely suited for investigating susceptibility to diabetes. This model offers several advantages over other experimental models of diabetes. First, it encompasses both sensitivity and resistance to a defined perturbation, whereas most other animal models represent sensitivity and lack a resistant counterpart [10]. Second, the model is non-obese, eliminating obesity as a confounding variable. Third, diabetes is diet-induced rather than spontaneous, enabling the incorporation of multiple control groups. Finally, the strains are genetically and phenotypically homogeneous, having undergone > 100 generations of brother–sister inbreeding since the model’s inception.

In designing the study, we reasoned that the most informative approach to examining the role of the gut microbiome in mediating sensitivity to diabetes in CDs/y and resistance to diabetes in CDr/y was to characterize and compare the microbial content of each strain in young, metabolically healthy animals prior to exposure to DD. Our analyses demonstrate that the gut microbial composition in early life is associated with divergent diabetes susceptibility, as both α- and β-diversity analyses consistently distinguished between the two strains. At 8 weeks of age, CDs/y exhibited higher α-diversity than CDr/y, and each strain maintained distinct community structures at both 8 and 16 weeks. Although reduced α-diversity is often associated with established metabolic disease, the higher diversity in CDs/y likely reflects differences in host–microbiome equilibrium rather than microbial “health” per se [11]. In this context, increased microbial diversity may indicate a more permissive gut ecosystem, allowing colonization by taxa that predispose to adverse metabolic responses upon dietary challenge. Thus, the microbiome configuration observed in CDs/y may confer vulnerability rather than protection, underscoring the need to distinguish microbial features associated with disease susceptibility from those associated with disease development.

We identified strain-specific microbiomes, seven genera enriched in CDs/y and nine genera enriched in diabetes-resistant CDr/y. In CDs/y, several of the enriched genera, including *UCG-010*, *Staphylococcus*, and *Vagococcus*, have previously been associated with diabetic phenotypes or disease progression, although not with diabetes sensitivity per se [12,13,14]. Notably, *p-2534-18B5_gut_group*, *WCHB1-41*, and *Clostridia_vadinBB60_ group* have not previously been linked to diabetes, and their enrichment in young, metabolically healthy but diabetes-sensitive animals suggests a potential role in conferring susceptibility. In CDr/y, in contrast, several of the enriched genera, including *Subdoligranulum*, *Oscillibacter*, *Olsenella*, *Marvinbryantia*, *Lachnoclostridium*, *Holdemanella*, and *Fusicatenibacter*, have been associated with favorable metabolic profiles, SCFA production, or improved glucose homeostasis [15,16,17,18,19,20,21,22]. Collectively, these findings suggest that resistance to diabetes is associated with a microbiome configuration enriched in metabolically supportive and SCFA-related functions, potentially enhancing resilience to a diabetogenic dietary challenge.

As our experimental design equated environmental conditions across all groups during the initial phase of the study, the strain-specific differences observed in gut microbial composition can be attributed to host genetic background. These findings highlight a substantial role for host genetics in shaping the gut microbiome, consistent with previous reports demonstrating that genetic variation influences microbial diversity and taxonomic composition, and that host–microbiome genetic interactions yield functional insights [23]. Our data support a model in which host genetics influences diabetes risk through two complementary mechanisms: directly, by regulating pathways involved in glucose metabolism [4], and indirectly, by shaping gut microbial communities. Genera enriched in diabetes-sensitive animals may promote metabolic perturbations or low-grade inflammation [24], whereas genera enriched in diabetes-resistant animals may confer protection through SCFA production, immune modulation, or enhanced barrier function [24,25]. These findings have translational relevance, supporting the potential for early-risk stratification during health using microbiome-based hypothesis-generating signatures and for the development of microbiota-targeted interventions tailored to genetically defined susceptibility profiles.

Having established an association between gut bacterial composition and susceptibility to diabetes, we next investigated whether this relationship is causal. Co-housing, which enabled partial passive microbial exchange between strains, resulted in attenuation of the diabetic phenotype in diabetes-sensitive animals, while the resistant phenotype remained unchanged. Interestingly, the asymmetric response mirrors our previous findings in this model, in which transfer of protective quantitative trait loci from resistant to sensitive animals mitigated diabetes, whereas transfer in the opposite direction had no effect [26]. Together, these observations support a causal contribution of the gut microbiome to diabetes susceptibility, reinforcing the concept that microbial composition actively modulates sensitivity and resistance to diet-induced diabetes. Co-housing also allowed us to address the magnitude of the gut microbiome’s contribution to diabetes susceptibility. Based on AUC measurements, co-housing was associated with a ~10% reduction in diabetes sensitivity. This quantitative effect indicates that the gut microbiome contributes meaningfully, albeit partially, to diabetes susceptibility, which is also shaped by genetic and other host-related factors. The “clinical” translation of such reduction in the susceptibility to diabetes following modulation of the microbiome suggests potential novel therapeutic targets and supports the development of microbiome-based intervention, but with no assurance of a “clinically” beneficial effect.

The secondary objective of this study was to explore the contribution of the gut microbiome to the development of diabetes in the Cohen rat model and determine whether its bacterial composition was distinct from that associated with susceptibility to diabetes. To achieve this aim, we compared the gut microbial composition at 8 and 16 weeks of age in animals fed DD or RD and studied microbiome changes at 16 weeks along with the development of diabetes in DD-fed CDs/y. We found that in addition to the genetic background which determines development of or lack of development of diabetes, the gut microbiome composition was strongly influenced by diet and age/time. Diet exerted a profound effect on the gut microbial composition across all groups. In CDs/y, however, we were unable to disentangle the effects of diet on the microbial composition of the gut from those of established diabetes. By 16 weeks, each group exhibited a distinct gut microbiome profile, suggesting that interaction between genetic background and diet contributes to the emergence of a diabetes-promoting microbiome in CDs/y and a diabetes-resistant microbiome in CDr/y.

In DD-fed CDs/y that developed diabetes, we identified genera associated with the development of diabetes that were distinct and markedly different from those associated with susceptibility to diabetes. These included *Victivallis*, *Aerococcus*, *Psychrobacter*, *Family_XIII_AD3011_group*, *Staphylococcus*, *Coriobacteriaceae_UCG-002*, *Chlamydia*, *Escherichia–Shigella*, and *Allobaculum*. Several of these taxa have been previously linked to diabetic phenotypes or metabolic dysfunction, including *Victivallis*, *Aerococcus*, *Psychrobacter*, *Staphylococcus*, *Coriobacteriaceae_UCG-002*, *Escherichia–Shigella*, and *Allobaculum* [13,14,27,28,29,30,31]. Notably, *Chlamydia*, a pro-inflammatory intracellular pathogen known to exacerbate insulin resistance, is implicated here for the first time in diabetes in a microbiome context. In contrast, CDr/y exhibited enrichment of genera associated with resistance to diabetes, including *Anaerotruncus, ASF356, Mucispirillum*, *Desulfovibrio*, *Lachnoclostridium*, *Dorea, Colidextribacter*, *Lactococcus*, *Collinsella*, *Holdemanella*, *Parasutterella*, *Sellimonas*, *Alloprevotella*, *Blautia*, and *Bacteroides*. Several of these taxa have been linked to favorable metabolic traits, including SCFAs, improved glucose metabolism, or restoration of intestinal homeostasis [17,32,33,34]. Reduced abundance of fermentative taxa such as *Anaerotruncus* and ASF356 has been reported in diabetic states, suggesting that their enrichment may contribute to metabolic resilience [20,35]. While some genera showed context-dependent associations [36], the overall microbial profile in CDr/y was enriched for taxa implicated in metabolic support and gut homeostasis.

Several limitations of this study should be acknowledged. First, only male rats were included, leaving the potential influence of sex on microbiome-mediated susceptibility to diabetes unresolved. Second, microbiome profiling was performed using 16S rRNA gene sequencing, which limits taxonomic resolution, may introduce PCR-related bias, provides relative rather than absolute abundance estimates, and offers limited functional insight. Finally, this study did not include functional validation of specific microbial taxa with respect to their role in susceptibility to or development of diabetes in this model. As the present work focused primarily on taxonomic composition, future studies incorporating metagenomic and metabolomic approaches will be important for elucidating the functional mechanisms underlying microbiome-associated diabetes susceptibility.

In summary, our findings position the gut microbiome as a dynamic interface between genetic predisposition, environmental exposures, and metabolic health. Using a uniquely informative animal model, the Cohen rat, we demonstrate that gut microbial composition is clearly associated with inherent susceptibility to diabetes and is distinct from that associated with the development of the disease. Our co-housing experiment mainly established evidence of association supporting causality. These results support the concept that distinct microbial signatures present during metabolic health may serve as early indicators of diabetes risk and highlight the microbiome as a potentially modifiable target for prevention or intervention strategies tailored to genetically defined susceptibility profiles.

## 4. Materials and Methods

### 4.1. Animals and Housing

Cohen diabetic rats were obtained from the Israeli Rat Genome Center at the Barzilai University Medical Center (Ashkelon, Israel).

Only male animals were used to reduce biological variability related to sex hormones, which are known to influence glucose metabolism and gut microbiome composition, and to maintain consistency with the established characterization of diabetes susceptibility in this model. A separate study in female animals is planned.

Animals were housed under standardized conditions with a 12 h light/dark cycle and ambient temperature maintained at 22–25 °C. Rats were kept in standard cages with identical bedding and environmental enrichment and had ad libitum access to food and water unless otherwise specified. Housing conditions were identical across strains and experimental groups to minimize environmental effects on gut microbiome composition.

For experiments involving microbial cross-transfer, animals were co-housed as described in the relevant experimental sections. All other husbandry practices were identical between groups.

### 4.2. Ethics Approval

All experimental procedures were conducted in accordance with the American Physiological Society guidelines for the care and use of laboratory animals and with the principles of the Israeli Ministry of Health Council for Animal Experimentation. The study protocol was approved by the Central Committee for Animal Experimentation of the Israeli Ministry of Health (approval number: NPC-BM-IL-2307-442-2).

### 4.3. Diet

Animals received regular diet (RD) by default, consisting of standard rodent chow (Sniff Spezialdiäten GmbH, Soest, Germany). RD contained 21% protein, 60% carbohydrates, 5% fat, and 0.45% NaCl and was provided from a single production batch throughout the study to minimize diet-related variability in gut microbiome composition.

To induce the diabetic phenotype, animals were provided a diabetogenic diet (DD) which was custom-prepared and composed of 18% copper-free β-casein, 72% sucrose, 4.5% butter, 0.5% corn oil, and 5% Salt No. II USP, supplemented with water- and fat-soluble vitamins. The diet was prepared according to established specifications for the Cohen diabetic rat model and supplied consistently across experimental groups.

When maintained on RD, both CDs/y and CDr/y remain metabolically normal. In contrast, following exposure to DD, CDs/y reproducibly develop impaired glucose tolerance and overt diabetes within approximately four weeks, whereas CDr/y remain metabolically normal, as previously described in this model [4]. In the present study, dietary exposure and metabolic phenotyping followed these established timelines.

Animals receiving RD had ad libitum access to tap water. Animals receiving DD were provided copper-free drinking water generated by reverse osmosis, as copper restriction in both diet and drinking water is a prerequisite for the development of the diabetic phenotype in CDs/y [4]. Water availability and handling were identical across experimental groups.

Dietary conditions, including diet composition, batch consistency, and timing of dietary transitions, were standardized across strains and experimental groups to minimize confounding effects on gut microbiome composition. Aside from the defined dietary interventions, no additional nutritional manipulations were introduced.

### 4.4. Study Design

We conducted two complementary sets of experiments to investigate the role of the gut microbiome in diabetes susceptibility and development, using the Cohen diabetic rat model.

#### 4.4.1. Experiment 1

To examine the association between the gut microbiome and diabetes susceptibility, we characterized and compared the metabolic phenotype and gut microbiome composition in young, metabolically healthy CDs/y and CDr/y maintained on RD. To assess the association with diabetes development, we subsequently studied the same groups of animals after eight weeks of feeding with DD or RD.

Study groups: We studied four groups of rats (n = 9 per group):

Group 1: CDr/y maintained on RD
Group 2: CDs/y maintained on RD
Group 3: CDr/y maintained on DD
Group 4: CDs/y maintained on DD

**Figure 8:**
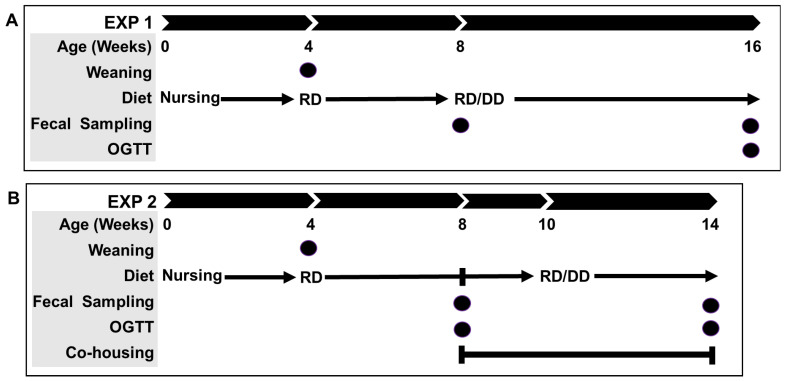
Timeline of experiments 1 (**A**) and 2 (**B**). RD, regular diet; DD, diabetogenic diet; OGTT, oral glucose tolerance test.

Study timeline (Figure 8A): After four weeks of maternal feeding, pups were weaned and maintained on RD. At eight weeks of age, we assigned the animals to the study groups. For the subsequent eight weeks, groups 1 and 2 continued on RD with tap water, whereas groups 3 and 4 received DD with copper-free water. We provided food and water ad libitum throughout the study. We euthanized the animals at 16 weeks of age.

Metabolic phenotyping: After eight weeks of RD or DD, we determined the phenotype of the animals using an oral glucose tolerance test (OGTT). Following an overnight fast (15 h), we measured baseline glucose from tail venous blood using a standard glucometer (time 0). We then gavaged the animals with a glucose solution (3.5 g/kg body weight, dissolved in water). We measured glucose levels at 15, 30, 60, 120, and 180 min and calculated the area under the OGTT curve (AUC).

Fecal microbiome collection: We collected fecal samples at two time points: (1) at the start of the study (8 weeks of age), when all animals were metabolically healthy, and (2) at the end of the study (16 weeks of age), after eight weeks of feeding with RD or DD, when diabetes had developed in CDs/y. We gathered fresh fecal pellets immediately upon defecation, placed them into sterile tubes, snap-froze the tubes in liquid nitrogen, and stored them at −80 °C until further processing.

#### 4.4.2. Experiment 2

To provide supportive evidence for the functional relevance of the microbiome–phenotype association, we co-housed CDs/y and CDr/y to allow passive cross-colonization of the gut microbiota. The effect of co-housing on the development of the diabetic phenotype was subsequently assessed in both strains.

Study groups: We studied six groups of animals: 4 single-housed control groups (n = 6 per group) and 2 co-housed experimental groups (Mix; n = 8 per group):

Group 1: CDr/y, single-housed, maintained on RD
Group 2: CDs/y, single-housed, maintained on RD
Group 3: CDr/y, single-housed, maintained on DD
Group 4: CDs/y, single-housed, maintained on DD
Group 5: CDr/y, co-housed with CDs/y (Mix), maintained on DD
Group 6: CDs/y, co-housed with CDr/y (Mix), maintained on DD

*Study timeline* (Figure 8B): We weaned all animals at 4 weeks of age and maintained them on RD for an additional 4 weeks. At 8 weeks of age, we allocated the animals to the study groups. Control groups (groups 1–4) were maintained separately as in Experiment 1 and provided with RD for 2 weeks, followed by either RD or DD for 4 weeks. In parallel, we co-housed experimental groups 5 and 6 (two CDs/y and two CDr/y per cage, four cages in total) and maintained them on RD for 2 weeks, followed by DD with copper-free water ad libitum for 4 weeks. We studied the metabolic phenotype using an OGTT at 8 weeks of age (baseline) and again at 14 weeks of age, after 6 weeks of co-housing and 4 weeks feeding with DD. We collected fecal samples for microbiome analysis immediately prior to the initiation of co-housing and at the end of the 6-week co-housing period.

### 4.5. Data Analyses

#### 4.5.1. Statistical Analysis

We are presenting data as mean ± SEM. We performed between-group comparisons using one-way ANOVA, with Fisher’s least significant difference (LSD) test for post hoc comparisons when appropriate. We set statistical significance at *p* < 0.05. We conducted the analyses using Statistica software (version 14.0.0.15).

#### 4.5.2. Analysis of Fecal Microbial Content

We determined the gut microbiome composition by extracting DNA from fecal samples using a QIAGEN DNA extraction kit using a standardized protocol for efficient lysis of bacteria, while minimizing host DNA contamination. We included extraction blanks and PCR negative controls to monitor background contamination. We amplified the V4 region of the 16S rRNA gene in a two-step PCR using primers 515F-Y and 806R-B, generating ~250 bp amplicons. We standardized sample collection, storage and DNA input across all replicates to ensure sample reproducibility and comparability. Libraries were indexed and sequenced on an Illumina MiniSeq platform (Illumina, San Diego, CA, USA), using 150 bp paired-end reads.

We merged reads using Pear [37], trimmed with Cutadapt, and processed using DADA2 [38] to infer amplicon sequence variants (ASVs). We removed chimeras using VSEARCH with the UCHIME de novo algorithm [39,40]. We performed taxonomic assignment in QIIME2 [41] using the SILVA 138 database [42]. We rarefied ASV tables to 12,000 reads per sample based on rarefaction analyses and assigned ASVs to organelles. We excluded ASVs with <10 total reads or present in fewer than two samples.

We assessed α-diversity using the Shannon, Simpson, Faith’s phylogenetic distance, and Chao1 indices and β-diversity using weighted and unweighted UniFrac, Bray–Curtis, and Jaccard distances. We used repeated-measures ANOVA for within-animal comparisons across time points, and linear model ANOVA for other group comparisons, with mixed linear models assessing interactions among experimental factors (Statsmodels). We visualized community differences by principal coordinates analysis and tested using PERMANOVA (adonis2) with the *vegan* package (version 2.7-2).

We evaluated the effects of strain, age/time, diet, and strain–diet interactions on gut bacterial diversity to distinguish signatures associated with diabetes susceptibility from disease development. We assessed differential taxonomic abundance at the genus level using ANCOM-BC2 (version 2.12.0), comparing profiles at 8 weeks of age on RD (susceptibility) and at 16 weeks after 8 weeks of DD or RD (development). We examined the effect of co-housing by comparing single-housed and co-housed animals within each strain under DD conditions using ANCOM-BC2 [19] before rarefaction, alongside evaluation of corresponding changes in metabolic and diabetic phenotypes.

## Figures and Tables

**Table 1:** In experiment 1, we explored the effects of strain, age/time and diet on α- and β-diversity. We compared the between strain effect at 8 (n = 18) and 16 weeks of age (n = 9), the within strain age/time effect at 8 (n = 9) and 16 (n = 9) weeks of age, and the within strain diet effect in each strain (n = 9 in each group). In experiment 2, we investigated the effects of co-housing on α- and β-diversity. We compared within strain the control (n = 6) to the MIX group (n = 8) and between strains the control (n = 6) to the MIX group (n = 8). Statistical analysis between groups was conducted using ANCOM-BC2.

		Experiment 1						Experiment 2			
		Strain Effect CDs/y vs. CDr/y on RD		Age/Time Effect 8 vs. 16 Weeks on RD Within Strain		Effect of Diet DD vs. RD Within Strain		Effect of CoH DD vs. Mix		Effect of CoH CDs/y vs. CDr/y	
Diversity	Index	Age 8 wks	Age 16 wks	CDs/y	CDr/y	CDs/y	CDr/y	CDs/y	CDr/y	DD	Mix
**α**	**Faith PD**	0.002	0.875	0.701	0.025	0.776	0.297	0.898	0.484	0.090	0.010
	**Shannon**	0.005	0.066	0.521	0.163	0.001	0.009	0.459	0.147	0.357	0.206
	**Simpson**	0.063	0.003	0.893	0.663	0.002	0.061	0.428	0.692	0.777	0.339
	**Chao1**	0.024	0.036	0.061	0.075	0.699	0.522	0.071	0.293	0.735	0.017
**β**	**Weighted Unifrac**	0.001	0.001	0.007	0.001	0.001	0.001	0.003	0.015	0.004	0.078
	**Unweighted Unifrac**	0.001	0.001	0.001	0.025	0.001	0.002	0.003	0.004	0.004	0.078
	**Bray-Curtis**	0.001	0.001	0.004	0.004	0.004	0.004	0.002	0.002	0.003	0.032
	**Jaccard**	0.001	0.001	0.001	0.001	0.001	0.001	0.001	0.001	0.003	0.051

## Data Availability

The datasets generated during and/or analyzed during the current study are available from the corresponding author upon reasonable request. The raw sequencing reads have been deposited at NCBI (ncbi.nlm.nih.gov) with the following accession numbers: PRJNA1418395 and PRJNA1420630.

## Supplementary Materials

The following supporting information can be downloaded at https://www.mdpi.com/article/10.3390/ijms27073160/s1.

## Abbreviations

ANCOM-BC2	Analysis of Compositions of Microbiomes with Bias Correction 2
ASVs	Amplicon Sequence Variants
CDr/y	Cohen Diabetic-Resistant
CDs/y	Cohen Diabetic-Sensitive
DD	Diabetogenic Diet
RD	Regular Diet
rRNA	Ribosomal RNA
SCFA(s)	Short-Chain Fatty Acid(s)
