# Supplementary material for: Gut Microbiome Signatures Distinguish Susceptibility from Disease Development in Type 2 Diabetes
Source: Int J Mol Sci. 2026 Mar 31;27(7):3160. doi: 10.3390/ijms27073160 (PMC13073554; doi:10.3390/ijms27073160)
Supplement: Supplementary file 1 [file ijms-27-03160-s001.zip › ijms-4173078-supplementary.pdf]

Table S1 – Supplement

Legend: Statistical parameters for taxa identified as significant by ANCOM-BC2 analysis as shown in Figures 6-8.

Susceptibility to Diabetes – Experiment 1 (Fig. 6)				
**Genus**	**beta (log fold change)**	**Standard error**	**q value (FDR)**	**95% Confidence Interval [Upper,Lower]**
Dubosiella	4.254	0.513	0.000	[3.249, 5.258]
p-2534-18B5_gut_group	3.617	0.444	0.000	[2.746, 4.488]
Vagococcus	1.215	0.592	0.000	[0.055, 2.376]
WCHB1-41	1.432	0.494	0.000	[0.463, 2.401]
Clostridia_vadinBB60_group	1.091	0.465	0.000	[0.181, 2.002]
Staphylococcus	0.699	0.491	0.000	[-0.262, 1.661]
UCG-010	0.100	0.353	0.000	[-0.592, 0.793]
Candidatus_Arthromitus	-2.852	0.567	0.000	[-3.963, -1.74]
Moryella	-1.352	0.585	0.046	[-2.498, -0.205]
Collinsella	-2.983	0.652	0.000	[-4.26, -1.706]
Mucispirillum	-1.803	0.735	0.035	[-3.243, -0.363]
Desulfovibrio	-1.815	0.723	0.030	[-3.231, -0.399]
NK4Agroup_214	-1.503	0.222	-2.168	[-1.939, -1.067]
Streptococcus	-2.439	0.802	0.007	[-4.011, -0.866]
Anaerobiospirillum	-6.473	0.608	0.000	[-7.664, -5.281]
Quinella	-3.076	0.646	0.000	[-4.342, -1.809]
Lachnospiraceae_UCG-001	-5.722	0.689	0.000	[-7.073, -4.372]
[Ruminococcus]_torques_group	2.115	0.722	0.010	[0.701, 3.53]
Bacteroidales_RF16_group	1.377	0.493	0.014	[0.411, 2.343]
g__Lachnospiraceae_NK4A136_group	2.374	0.687	0.002	[1.028, 3.719]
Enterococcus	1.936	0.808	0.040	[0.351, 3.521]
Prevotellaceae_UCG-003	1.857	0.587	0.005	[0.706, 3.007]
Anaerovibrio	-3.359	0.652	0.000	[-4.636, -2.081]
Escherichia-Shigella	-2.305	0.846	0.017	[-3.964, -0.646]
Helicobacter	-1.553	0.560	0.015	[-2.651, -0.456]
Chlamydia	2.533	0.863	0.010	[0.842, 4.224]
Blautia	-2.113	0.583	0.001	[-3.255, -0.971]
Rikenellaceae_RC9_gut_group	2.166	0.438	0.000	[1.306, 3.025]
Turicibacter	4.491	0.579	0.000	[3.356, 5.625]
Clostridium_sensu_stricto_1	3.444	0.913	0.001	[1.654, 5.234]
Roseburia	-1.643	0.725	0.050	[-3.064, -0.222]
Muribaculaceae	1.786	0.415	0.000	[0.974, 2.599]
Allobaculum	1.571	0.381	0.000	[0.825, 2.318]

Subdoligranulum	-1.275	0.393	0.000	[-2.045, -0.505]
Dorea	-1.505	0.428	0.000	[-2.344, -0.666]
Oscillibacter	-1.389	0.434	0.000	[-2.24, -0.539]
Olsenella	-2.145	0.481	0.000	[-3.088, -1.202]
Marvinbryantia	-2.404	0.550	0.000	[-3.482, -1.325]
Lachnoclostridium	-2.067	0.585	0.000	[-3.213, -0.921]
Shuttleworthia	-2.130	0.599	0.000	[-3.305, -0.955]
Holdemanella	-4.817	0.511	0.000	[-5.817, -3.816]
Fusicatenibacter	-6.141	0.418	0.000	[-6.96, -5.321]

Development of Diabetes - Experiment 1 (Fig. 7)				
**Genus**	**beta (log fold change)**	**Standard error**	**q value (FDR)**	**95% Confidence Interval [Upper, Lower]**
**CDs-DD-16**				
Allobaculum	-1.872	0.475	0.000	[-2.802, -0.942]
Escherichia-Shigella	-3.925	1.184	0.003	[-6.246, -1.604]
Chlamydia	-3.114	1.003	0.000	[-5.079, -1.149]
Coriobacteriaceae_UCG-002	-4.361	0.751	0.000	[-5.832, -2.889]
Staphylococcus	-3.566	0.881	0.000	[-5.293, -1.839]
Family_XIII_AD3011_group	-3.248	0.869	0.000	[-4.952, -1.544]
Psychrobacter	-1.543	0.537	0.000	[-2.596, -0.49]
Aerococcus	-1.557	0.575	0.000	[-2.685, -0.429]
Victivallis	-1.552	0.552	0.000	[-2.634, -0.47]
**CDr-DD-16**				
Bacteroides	-1.014	0.209	0.000	[-1.423, -0.605]
Blautia	-6.163	0.597	0.000	[-7.332, -4.994]
Alloprevotella	-1.899	0.386	0.000	[-2.656, -1.142]
Sellimonas	-1.550	0.204	0.000	[-1.95, -1.15]
Parasutterella	-2.553	0.688	0.001	[-3.901, -1.205]
Holdemanella	-4.592	0.412	0.000	[-5.399, -3.785]
Collinsella	-3.294	0.831	0.000	[-4.922, -1.666]
Lactococcus	-4.158	0.644	0.000	[-5.419, -2.896]
Colidextribacter	-2.999	0.859	0.000	[-4.682, -1.315]
Dorea	-0.803	0.644	0.000	[-2.066, 0.459]
Lachnoclostridium	-2.562	0.822	0.005	[-4.173, -0.951]
Desulfovibrio	-4.585	1.016	0.000	[-6.575, -2.594]
Candidatus_Stoquefichus	-0.737	0.755	0.000	[-2.217, 0.743]
Mucispirillum	-1.870	0.775	0.000	[-3.388, -0.351]
ASF356	-2.155	0.791	0.027	[-3.707, -0.604]
Anaerotruncus	-1.422	0.706	0.000	[-2.806, -0.038]
Mycoplasma	-1.240	0.532	0.000	[-2.283, -0.196]

Co-housing - Experiment 2 (Fig. 8)				
**Genus**	**beta (log fold change)**	**Standard error**	**q value (FDR)**	**95% Confidence Interval [Upper, lower]**
Staphylococcus	4.769	0.364	0.000	[4.056, 5.483]
Aerococcus	3.246	1.034	0.000	[1.22, 5.272]
Dubosiella	3.098	0.947	0.000	[1.241, 4.954]
Facklamia	2.369	0.928	0.000	[0.55, 4.189]
Lactococcus	3.406	0.747	0.000	[1.943, 4.87]
Vagococcus	2.389	0.959	0.000	[0.51, 4.268]
Gastranaerophilales	1.269	0.543	0.000	[0.206, 2.333]
Negativibacillus	0.930	0.288	0.009	[0.366, 1.494]
Alloprevotella	-1.251	0.344	0.003	[-1.924, -0.577]
Parabacteroides	-1.014	0.301	0.007	[-1.605, -0.424]
Enterococcus	-3.069	0.915	0.007	[-4.862, -1.276]
Anaerovibrio	-1.460	0.483	0.015	[-2.408, -0.513]
Prevotella	-3.744	1.006	0.003	[-5.715, -1.772]
Prevotellaceae_UCG-001	-3.596	0.791	0.000	[-5.146, -2.045]
Fusicatenibacter	-5.291	0.234	0.000	[-5.749, -4.832]
Anaerobiospirillum	-2.674	0.850	0.000	[-4.34, -1.009]
Anaerostipes	-1.621	0.944	0.000	[-3.471, 0.228]
Chlamydia	-1.426	0.857	0.000	[-3.107, 0.254]
Elusimicrobium	-1.326	0.791	0.000	[-2.876, 0.224]
